# The Impact of SARS-CoV-2 Pandemic on Antibiotic Prescriptions and Resistance in a University Hospital from Romania

**DOI:** 10.3390/antibiotics13060477

**Published:** 2024-05-23

**Authors:** Dana Carmen Zaha, Codrin Dan Nicolae Ilea, Florica Ramona Dorobanțu, Carmen Pantiș, Ovidiu Nicolae Pop, Dorina Gabriela Dascal, Cătălin Dorin Dorobanțu, Felicia Manole

**Affiliations:** 1 Department of Preclinical Disciplines, Faculty of Medicine and Pharmacy, University of Oradea, 1st University Street, 410087 Oradea, Romania; dzaha@uoradea.ro (D.C.Z.); dascal.dorinagabriela@didactic.uoradea.ro (D.G.D.); 2 Bihor Emergency Clinical County Hospital, 410087 Oradea, Romania; 3 Department of Biostatistics, Faculty of Medicine and Pharmacy, University of Oradea, 1st University Street, 410087 Oradea, Romania; 4 Department of Medical Disciplines, Faculty of Medicine and Pharmacy, University of Oradea, 1st University Street, 410087 Oradea, Romania; rdorobantu@uoradea.ro; 5 Department of Surgical Disciplines, Faculty of Medicine and Pharmacy, University of Oradea, 1st University Street, 410087 Oradea, Romania; carmen.pantis@didactic.uoradea.ro (C.P.); cdorobantu@uoradea.ro (C.D.D.); fmanole@uoradea.ro (F.M.)

**Keywords:** COVID-19, antimicrobial resistance, antibiotic consumption

## Abstract

This paper aimed to evaluate the effects of the COVID-19 pandemic on prescription rates and antibiotic resistance in a university hospital. A retrospective study was conducted on the medical records of patients admitted to the Bihor Emergency Clinical County Hospital in Romania in 2019 (pre-pandemic) and 2021 (during the pandemic period). We evaluated the antibiotic consumption index (ACI) and susceptibility rates. The overall percentage of antibiotic prescribing increased in 2021, while the total number of patients decreased. Genito-urinary, digestive, respiratory infections, heart diseases and wounds were the most common conditions for antibiotic prescriptions, but the number of them decreased in 2021. There was a decrease in the proportion of antibiotics from the Watch and Reserve class and an increase in the proportion of antibiotics from the Access class. Antibiotic use has been reduced despite an increase in the number of patients, with a high consumption in the Watch group in the ICU wards. By contrast, surgical wards had the highest rate of antibiotic prescriptions, but a decrease in the number of patients. The patients who were administered antibiotics were hospitalized for diagnoses other than COVID-19. Almost all prescribed antibiotics displayed decreasing sensitivity rates. The number of isolated ESKAPE pathogens, except for *Staphylococcus aureus* methicillin-resistant strains, were increased. Strategies to control antibiotic prescriptions and the spread of resistant pathogens should be improved.

## 1. Introduction

Antibiotics are prescribed in medical practice for prophylactic and therapeutic purposes for infections. The improper use (wrong dose or overdose) of antibiotics facilitates the development of antibiotic resistance in microorganisms, allergies, dermatological, hematological, renal and neurologic adverse events and causes side effects—mostly gastrointestinal: e.g., nausea, diarrhea, *C. difficile* infections [1,2,3,4]. Antimicrobial resistance (AMR) is a worldwide concern and public health problem that is exacerbated by the overuse of antibiotics, with a large economic burden on healthcare systems. Many reports have highlighted an impending rate of 10 million yearly deaths due to AMR by 2050, amounting to one death every three seconds. Otherwise, AMR was categorized as the top ten global public health threat by the World Health Organization in 2019, necessitating immediate interventions [5,6]. For these reasons, it is very important to know and apply rational drug use into clinical practice.

Rational antibiotic use is defined as taking antibiotics in accordance with the clinical needs of the patient, at appropriate doses, for sufficient time, at the lowest cost to themselves and society. Information concerning the indications and doses of antibiotics and prophylaxis is updated frequently and medical staff should follow the current guidelines, prescribe according to results from exploratory tests and attend education programs. Although the literature contains studies evaluating the patterns of prescription, knowledge and attitudes of medical staff with regards to antibiotic prescription, there are not enough studies in this field [7,8].

AMR has particularly grown in the last two decades, becoming an urgent threat to global health [9,10]. In hospitals and wards with a high rate of antimicrobial prescription such as intensive-care units and surgical wards, infections caused by difficult-to-treat bacteria are increasingly associated with elevated mortality and increasing hospital costs [11,12,13]. The European Centre for Disease Prevention and Control (ECDC) reported more than 670,000 infections and 33,000 deaths due to multi-resistant bacteria in the course of 2019 [14]. Antimicrobial stewardship (AMS) must improve the outcomes, quality and safety of patient care at the same time as preventing or controlling the spread of AMR. Ultimately, this consists of responsible antibiotic use and prescribing effective antibiotics to treat infections. According to many national action plans, the target is to reduce antimicrobial use, both for humans and animals, and this could start with a reduction in community antibiotic use [15,16]. To achieve this goal, better understanding of current antibiotic prescribing pattern is needed. However, the implementation of guidelines in primary care and hospitals has not been satisfactory in many countries [17]. Analyses of patterns of antibiotic prescribing involve the renewal of short-term antibiotic prescriptions for acute issues that exist beyond a single course of treatment, general practices and hospital prescribing habits, as well as additional infections that occur over a certain period. The reasons for antibiotic prescribing may not always be well documented, with up to half of antibiotic prescriptions being unrelated to any specific medical diagnosis recorded [18].

Coronavirus disease (COVID-19) is an infectious respiratory disease caused by the SARS-CoV-2 virus. Both COVID-19 and bacterial pneumonia share similar clinical features, but the COVID-19 pandemic has challenged the implementation of antimicrobial stewardship programs and the use of antimicrobials in clinical practice. On the other hand, the impact of the COVID-19 pandemic on antimicrobial resistance is still largely unknown; however, there is evidence that antimicrobial prescription habits have been profoundly affected [19]. According to the Center for Disease Control and Prevention (CDC), the COVID-19 pandemic is responsible for a 15% increase in AMR and related deaths in hospitals in 2020 [20]. Therefore, exploring data concerning the impact of the COVID-19 pandemic on antibiotic prescribing and AMS is an essential step for improving and updating existing policies.

The pandemic has triggered an unexpected crisis in health care systems for healthcare workers, patients and hospitals. Different bacterial infections occur in COVID-19 pandemic patients—particularly in patients with severe disease, with the majority being hospital-acquired co-infections [21]. The most frequent causal agents of hospital-acquired infections are a group of six virulent and resistant pathogens called ESKAPE pathogens, referring to *Enterococcus faecium, Staphylococcus aureus*, *Klebsiella pneumoniae*, *Acinetobacter baumannii*, *Pseudomonas aeruginosa* and *Enterobacter* species. The list of pathogens can be completed with *Escherichia coli* because of its importance as the main etiological agent of many infections and the reason for the prescription of many antibiotics. *Enterococcus* sp. includes *Enterococcus faecium* and *Enterococcus faecalis*, gram-positive commensals commonly found in the gut. Enterococci are associated with hospital-acquired infections including urinary and catheter-associated urinary tract infections, surgical site infections and bloodstream infections. According to the CDC, about 30% of all healthcare-associated enterococcal infections are resistant to vancomycin, resulting in reduced treatment options, and these infections were estimated to cause 5,400 deaths in 2017 in the United States [22].

Although these pathogens are frequently isolated from environments such as surface water, wastewater and soil, they are also involved in hospital-associated infections. In hospitalized critically ill COVID-19 patients, complications involving a secondary infection usually involve multidrug-resistant (MDR) bacteria, at a rate ranging from 30–50%, with the majority being respiratory and blood stream infections occurring a week after admission [23].

The aim of this paper was to investigate whether the prescription of antibiotics for specific infections caused by ESCAPE pathogens and antibiotic resistance in our hospital has changed during the COVID-19 pandemic, and to identify new trends in prescriptions that may be a consequence of the COVID-19 pandemic. The primary objective of the study was to evaluate the consumption of prescribed antibiotics and the clinical conditions associated with this, and the second was to compare the microbiological profile of the hospital before and during the COVID-19 pandemic.

## 2. Results

A total of 36,076 and 23,903 patients from 2019 and 2021, respectively, were admitted to the hospital. The overall number of antibiotic prescriptions was for 19,502 patients in 2019 and for 14,660 patients in 2021, respectively, showing an increase in 2021 compared to 2019 (61.33% versus 54.06%, *p* < 0.00001) while the total number of patients decreased. The general demographic characteristics of the study population are summarized in Table 1.

In 2019, 11,630 (59.63%) of patients were female, increasing to 14,255 (60.76%) in 2021. Similarly, in 2019, 7872 of patients (40.37%) were males, with a slight decrease to 5753 (39.24%) in 2021.

**Figure 1:**
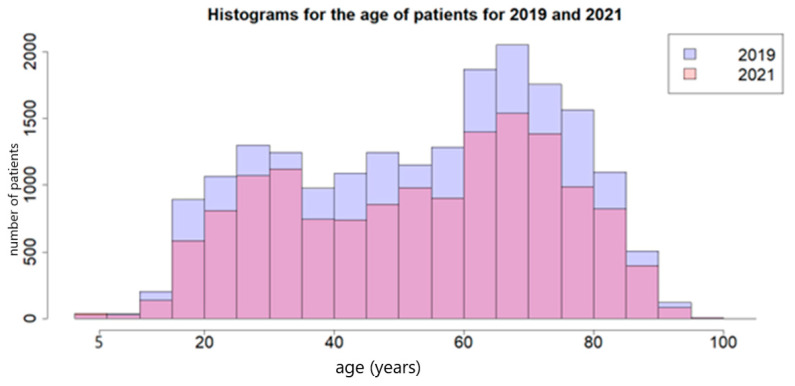
Age of patients in 2019 and 2021.

The age of patients admitted in 2019 and 2021 ranged from 5 to 98 years, with an average of around 54, but the difference in the overall age category was not statistically significant (Figure 1). The age category of 60–80 encompassed the greatest number of admissions in 2019 and 2021. However, the age of the patients was younger in the case of patients hospitalized in 2021.

The outcome documented a doubling of the mortality rate in 2021 (4.63% versus 8.04%, Pearson’s Chi-squared test, *p* < 0.05), but this interpretation must be performed carefully in terms of causes of deaths. We explored conditions for which antibiotics are prescribed, and we classified them as follows: Genito-urinary, digestive, respiratory infections, heart diseases and wounds were the most common conditions for antibiotic prescriptions, but the number of them decreased in 2021. Conversely, the number of surgical complications treated with antibiotics and other viral etiologies increased in 2021 (Table 2).

A total number of 911 patients were diagnosed and hospitalized for COVID-19, and half of them (584 patients) were treated with antibiotics in 2021. Out of the 584 patients, 475 presented with COVID-19 and bacterial infections. If we take into account these 475 COVID-19 patients treated with antibiotics and refer to the total number of patients in 2021, it turns out that the patients who were administered antibiotics were hospitalized for diagnoses other than COVID-19. However, a significant number of patients, regardless of the evaluated year, were prescribed antibiotics without having a specific reason (Table 2, other conditions without infectious disease), but the number of them decreased.

Most of the patients who were prescribed antibiotics were admitted to the surgery and medical wards, but the number of patients decreased in 2021. Instead, the number of patients who were prescribed antibiotics after being admitted to the ICU in 2019 was the lowest; this number later increased, surpassing that of patients admitted to medical wards in 2021 (Table 3).

Table 4 documents that surgical wards had the highest rate of antibiotic prescriptions, as expressed as ACI, followed by medical wards with a significant increase in 2021, while the consumption of antibiotics in the ICU decreased slightly. The prescription of antibiotics increased five times in the surgical wards and three times in medical wards, but statistical significance was obtained only for surgical wards and the ICU (*p* < 0.00001, Table 4).

Most of the antibiotics prescribed showed increases in prescribed doses in 2021, but statistical significance was obtained only for ceftriaxone, cefixime, cefuroxime, metronidazole, ampicillin, amikacin, gentamicin, rifaximin and clindamycin. On the other hand, teicoplanin and benzylpenicillin were prescribed less often in 2021, while cefaclor and cefazolin were no longer prescribed at all.

The global evaluation of the consumption of antibiotics according to the AWaRe classes showed a high consumption of antibiotics in the Watch group, followed by the Access group. Worth noting is the decrease in the proportion of antibiotics from the Watch and Reserve class and the increase in the proportion of antibiotics from the Access class (*p* < 0.00001) when comparing 2021 to 2019 (Table 5).

Regarding the Access group of antibiotics, Metronidazole (ACI: 1216.38 vs. 104.81, *p* < 0.00001), amikacin (ACI: 308.83 vs. 34.24, *p* < 0.00001), ampicillin (ACI: 297.06 vs. 132.46, *p* < 0.00001), gentamicin (ACI: 116.54 vs. 89.45, *p* < 0.00001) and clindamycin (ACI: 89.07 vs. 42.85, *p* < 0.01) consumption was increased, while benzylpenicillin consumption (ACI: 2.38 vs. 22.71, *p* < 0.00001) was decreased during the pandemic period. For the rest of the Access class antibiotics, increases were recorded in 2021, but without statistical significance (Table 6).

The prescriptions of each antibiotic from the Watch group were increased in 2021, except for teicoplanin (ACI: 20.06 vs. 22.20, *p* < 0.00001). Significant increasing doses occurred for ceftriaxone (ACI: 1438.52 vs. 450.90, *p* < 0.05), cefuroxime (ACI: 449.36 vs. 255.52, *p* < 0.00001), cefixime (ACI: 434.70 vs. 9.95, *p* < 0.00001) and rifaximin (ACI: 123.53 vs. 67.63, *p* < 0.00001; Table 7).

Without reaching statistical power, significant increases in the doses of antibiotics prescribed were also recorded for those in the Reserve group, but they remained the lowest prescribed doses (Table 8).

The most prescribed antibiotics belong to the cephem class, presenting a tripling of the prescribed doses (ACI: 2457.28 vs. 767.53, *p* < 0.001), but imidazole showed the greatest increase (ACI: 1216.38 vs. 104.81, *p* < 0.00001). Penicillins (ACI: 482.50 vs. 207.98, *p* < 0.00001), rifamycins (ACI: 123.53 vs. 67.63, *p* < 0.00001), lincosamides (ACI: 89.07 vs. 42.85, *p* < 0.01) and glycopeptides (ACI: 77.69 vs. 40.42, *p* < 0.01) showed almost double increases in prescriptions. During the evaluated period, no antibiotics were prescribed from the classes of folate pathway inhibitors and nitrofurans (Table 9).

In relation to the consumption of antibiotics we explored the sensitivity rate of them; amikacin, piperacillin/tazobactam, ceftazidime/avibactam, ceftazidime, cefepime, cefotaxime, cefixime, trimethoprim/sulfamethoxazole, imipenem, meropenem, ertapenem, benzylpenicillin, azithromycin, ciprofloxacin and levofloxacin showed significant decreasing sensitivity rates. Conversely, sensitivity rates were increased for fosfomycin, vancomycin, clindamycin, colistin and ofloxacin (Table 10).

We explored the isolated ESKAPE pathogens and their resistance phenotype. There was an increase in the number of isolated *Acinetobacter baumannii*, *Pseudomonas aeruginosa* and *Klebsiella pneumoniae* strains, as well as those expressing a resistance phenotype—particularly to carbapenems (*p* < 0.00001) during 2021. The number of isolated *E. coli* and *Enterobacter* sp. strains decreased slightly, but the number of ESBL and carbapenem-resistant increased significantly. There was a decrease in the number of isolated *Staphylococcus aureus* strains, as well as MSRA ones (Table 11).

## 3. Discussion

In this study, we used data from medical records and reports from some hospital departments to explore whether and how the COVID-19 pandemic has influenced antibiotic consumption and the microbiological profile. For this purpose, we evaluated 2019 as a pre-pandemic year and 2021 as a year during the pandemic.

The widespread transmission of SARS-CoV-2 infections has been a challenge for healthcare facilities, changing some practices, increasing the number of admissions and exerting pressure on personnel and medical facilities [24,25]. In the current study an increasing trend was observed, both in terms of antibiotic consumption and the number of patients treated with antibiotics, although the number of patients decreased. Total antibiotic consumption expressed as ACI before the pandemic period was 1075.07 and the total use of antibiotics during the pandemic was 2548.57, representing a significant increase. Most studies showed increasing antibiotic prescriptions during the COVID-19 pandemic [26,27], but there are other studies describing a reduction in their prescription [28,29]. Many factors may be considered to explain the increased prescription of antibiotics during the COVID-19 pandemic, such as a misunderstanding of how to treat these infections, inexperience, hospital overcrowding, the limited number of medical staff versus the number of patients, changes in the antimicrobial stewardship team’s activity and a lack of initial therapeutic protocols. This emphasizes the importance of antimicrobial stewardship in controlling and optimizing antibiotic use in hospitals, including in emergencies and in situations like the COVID-19 pandemic [30]. Age does not appear to impact antibiotic prescribing patterns significantly, but gender does. It seems that female patients are administered antibiotics more frequently regardless of the pandemic context [31].

Reported mortality rate represents a partial count of the total deaths from the COVID-19 pandemic, but the distribution and significance of other causes of death have changed because of many reasons (social, health politics, economic, behavioral). The reliability of reported deaths varies greatly between countries, locations and hospitals, and over time and it is difficult to frame COVID-19 as the main cause. When compared with the pre-pandemic period, the mortality rate of nearly 8.04% during the pandemic showed an increasing rate, as international statistics and other studies have shown [32,33,34]. There could be many explanations for this, such as aggravated chronic conditions or presentation to a hospital during the complication phase with or without COVID-19 infection.

According to current guidelines, the prescription of antibiotics is recommended under specific clinical conditions (infections) or for prophylactic purposes. The significant clinical conditions for which increasing doses of antibiotics were administered were respiratory conditions, surgical complications, genito-urinary infections and wounds while digestive conditions were decreased. In our study, the number of patients who were administered prophylactic antibiotics increased, but the number of patients decreased. In addition, there was a significant number of patients who were prescribed antibiotics without having a specific reason, but this number decreased in 2021. The result is an excess of prescriptions and the need for intervention from the antibiotic stewardship committee, for regular updates of clinical practice guidelines—especially for empirical treatment antibiotics—the following of these guidelines and education [35]. Stewardship program interventions must improve the quality of care through the improvement of prescription decisions in hospital settings.

A total of 911 patients with COVID-19 were hospitalized, and half were treated with antibiotics, presenting with bacterial complications (52.14%). These patients presented also with advanced age and comorbidities that required antibiotic treatment. The proportion of patients treated with antibiotics that had COVID-19 was 3.24%, which shows that antibiotics were mainly prescribed for conditions other than COVID-19. More than half of the patients with COVID-19 and those of older age admitted into the ICU were in critical condition or died. The number of patients who were administered antibiotics increased in the ICU in 2021, but the consumption of antibiotics in the ICU decreased in 2021 (*p* < 0.00001). This can be explained by the administration of antibiotics only to patients who had a documented bacterial infection, regardless of whether they were COVID-19 patients or not. By contrast, surgical wards had the highest rate of antibiotic prescriptions and a reduction in the number of patients, similar to other studies [36]. This aspect can be explained by the doubling of the number of patients with surgical complications. Similar aspects have also been described in the medical ward, i.e., a reduction in the number of patients with a significant increase in the consumption of antibiotics, expressed as ACI. The explanations for this could be related to the patients or to prescribing habits. As the patients presented late, in the complications phase, the prescribing doctors applied the strongest treatments, not necessarily following the guidelines—including the guidelines for COVID-19 management.

The World Health Organization (WHO) has released the WHO AWaRe (Access, Watch, Reserve) classification of antibiotics in addition to the model list of essential medicines [37]. They are very useful for guidance on the use of antibiotics, taking into consideration the risk of antimicrobial resistance development, and they could be used by many hospitals. The group of the Watch antibiotics have a higher potential for developing antimicrobial resistance and their use should be carefully monitored. Although the prescription of these antibiotics was shown to be higher in 2019, a decrease for ceftriaxone, cefuroxime, cefixime and rifaximin was still observed during COVID-19 in our study. Reserve group of antibiotics are last-option antibiotics that should only be used for the treatment of severe infections caused by multi-drug-resistant pathogens. In our study, we observed a decreased trend for the use of colistin, tigecycline, linezolid, ceftazidime/avibactam and imipenem/cilastatin/relebactam. Access antibiotics are antibiotics with fewer side-effects and a lower potential for the development of antimicrobial resistance, and they should be used for the empiric treatment of most common infections. In the present study, the trend for metronidazole, amikacin, ampicillin, gentamicin and clindamycin use during the COVID-19 pandemic increased, and this is a good direction to be continued.

A study reporting reduced hospitalizations due to airway respiratory tract infections during the COVID-19 pandemic led to a decrease in the consumption of antibiotics, especially for penicillins and beta-lactamase inhibitors [38]. In our study, the consumption of cephalosporins, imidazoles, penicillins, rifamycins, lincosamides and glycopeptides, ex-pressed as ACI, increased in 2021 compared to 2019, and only two antibiotics showed decreases in 2021—benzylpenicillin and teicoplanin, as other studies have reported [39,40,41]. Other studies reported a decrease in cephalosporin prescriptions during the COVID-19 pandemic, along with the prescription of macrolides, lincosamides and quinolones [42,43].

Despite insufficient statistical power, we observed an increase in quinolone prescriptions, contrary to many other studies showing a reduction in quinolones throughout the COVID-19 pandemic; the prescription of these antibiotics should be monitored and rationalized for specific indications for children and adults [39,44]. These differences in results could be explained if fluoroquinolones are used as empirical therapies rather than for a single specific antimicrobial therapy against pathogenic germs [41].

From the imidazole class, only metronidazole was prescribed, showing the most significant increase in the prescription rate—practically 12 times more. Coronavirus disease 2019 involving the upper respiratory tract followed by severe pneumonia, respiratory distress and/or even death has rapidly emerged as a global pandemic. Many studies have found higher blood levels of some pro-inflammatory cytokines during this infection. In the same context, there are studies showing that metronidazole could decrease the levels of some cytokines—especially for interleukin 8, 6, 1B, 12, 1α, tumor necrosis factor (TNF) α, interferon γ, as well as the levels of C-reactive protein (CRP) and neutrophil counts. An increased consumption of metronidazole has been recorded, prescribed more often for non-COVID patients [45].

ESKAPE pathogens are involved in the increases in morbidity and mortality related to antibacterial resistance. During COVID-19, co-infections—especially with Gram-negative ESKAPE bacterial and fungal were more frequent in patients with severe COVID-19 symptoms than in patients with milder symptoms [46]. *A. baumannii*, *K. pneumoniae* and *Enterobacter* spp., are the most prevalent strains in nosocomial pneumonia, complicating the management of COVID-19 patients who need to be ventilated in the intensive care unit (ICU), and all these pathogens were increased in number during 2021, including increases in carbapenem-resistant and ESBL pathogens. Among the patients with bacterial infections, the majority had Gram-negative infections and/or mixed infections with Gram-positive and Gram-negative pathogens, regardless of the evaluated year. Among the Gram-negative bacteria, *K. pneumoniae* was the predominant pathogen, followed by *A. baumannii*. In one study, it was reported that during COVID-19, *P. aeruginosa* was the main pathogen associated with ventilator-associated pneumonia in critical patients, but in our study, it represented only the third most common etiology after *A. baumannii* and *K. pneumoniae*. Carbapenems are considered the most appropriate agents to treat Gram-negative infections, but these strains displayed high levels of resistance to carbapenems in 2021. Due to these resistance mechanisms, polymyxins are considered the best option for the treatment of CR-*A. baumannii* and CR-*P. aeruginosa* infections [47].

Methicillin-resistant *Staphylococcus aureus* (MRSA) is one of the major pathogens responsible for bloodstream infections and multi-drug resistance (MDR), commonly associated with hospital-acquired infections. There are studies that have reported whether the prevalence rates of MRSA have decreased or not during COVID-19 pandemic, but variation between hospitals, population and geography are described [48,49]. Our findings are in accordance with these studies, but the sensitivity rates of effective antibiotics against MRSA have increased (vancomycin, clindamycin, teicoplanin) and are less prescribed.

The main limitations of our study are as follows: First, we explored a single hospital and its prescription practices, the susceptibility rates of the antibiotics used and the resistant pathogen profile. Second, we were unable to examine the prescriptions case-by-case or explore each clinical situation or other factors involved in therapeutic decisions. Prolonged hospitalization and immunosuppression during COVID-19 exposed those patients to infectious complications with resistant pathogens and increased antibiotic prescriptions.

## 4. Materials and Methods

This retrospective study compared data concerning antibiotic use by analyzing the medical reports of patients admitted to the Bihor Emergency Clinical County Hospital, Romania between 1 January and 31 December of 2019 (before the COVID-19 pandemic) and 1 January–31 December of 2021 (during the COVID-19 pandemic year). This is a teaching multidisciplinary hospital with more than 500 beds and a large number of pathologies, grouped in this study into the Intensive Care Units (ICUs), surgical and medical wards.

Data was collected from the patients’ electronic medical records. The study population included all inpatients registered in two cohorts corresponding to 2019 and 2021, respectively, who received antibiotics in different departments of the hospital. Age, gender and outcomes were extracted and analyzed. Data on antibiotic prescriptions were extracted from the pharmacy’s reports.

The study of antibiotic prescriptions was performed using the Anatomical Therapeutic Chemical Classification System (ATC/DDD, 2016) developed by the WHO Collaborating Centre for Drug Statistics Methodology, ATC/DDD Index 2022 [50]. First, antibiotic prescription patterns were expressed in grams, and then we calculated the antibiotic consumption index (ACI) using the following formula:

ACI = (total dose of antibiotic (grams)/DDD × total patient days) × 100 for each antibiotic

We classified antibiotics into classes and WHO AWaRe (Access, Watch, Reserve) anti-biotics [51].

We considered genito-urinary, digestive and respiratory infections, heart diseases and wounds as the main diagnoses for which antibiotics were prescribed.

Antibiotic susceptibility testing was performed with a Vitek 2 system according to the recommendations of the European Committee on Antimicrobial Susceptibility Testing (EU-CAST) criteria [52].

Statistical analysis was performed using the R program (https://www.r-project.org/ (accessed on 4 January 2024)), version 4.3.1. Descriptive statistics were used to summarize the baseline characteristics of the study population, including age and sex. To verify the statistical significance of the obtained results, the Chi-square test (χ2) and the *t*-test were used. The confidence interval was set at 95%, with a statistical significance threshold of 0.05.

We calculated antibiotic sensitivity rates by considering all isolated strains per year, and a minimum of 30 from each isolate by using WHONET 2023 software. For 2019, we performed this evaluation on 5987 strains and on 6212 strains in 2021. Results were expressed as %R, %I, %S and %R 95% confidence intervals.

Individual patients’ written informed consent was obtained at admission. This study was approved by the Ethics Committee of the County Clinical Emergency Hospital of Oradea and is in full agreement with the World Medical Association Declaration of Helsinki.

## 5. Conclusions

The World Health Organization (WHO) has recognized that the inappropriate and irrational use of antibiotics has been followed by antibiotic resistance and that the COVID-19 pandemic has challenged the management of patients, antibiotic use and the surveillance of some critical categories of bacteria from the point of view of antibacterial resistance development. It is well known that the inappropriate use of antibiotics represents the major cause of AMR onset, and that this can be prevented. In addition, in cases of the weakening of immune system defenses occurring during viral or bacterial infections, as well as other immune diseases, antibiotic consumption and resistance trends must be controlled to anticipate subsequent changes. Our results show that patients who were administered antibiotics were hospitalized for diagnoses other than COVID-19. The escalation in antibiotic prescription among hospitalized patients and the increase in antimicrobial resistance at the same time during the COVID-19 pandemic had a local impact on antibiotic consumption and antimicrobial resistance rates in 2021. However, the isolation of resistant isolates in the hospital setting emphasizes the need to apply and update antimicrobial stewardship programs. Antibiotic prescriptions should follow current guidelines and there is a continuous need for education in the correct diagnosis and treatment of infectious diseases, as well as rational drug use. Continued research efforts and strategies to control the spread resistant pathogens are needed to address this public health threat.

## Figures and Tables

**Table 1:** Distribution of the demographic characteristics of patients admitted before and during the COVID-19 pandemic.

Patient Characteristics	2019 N (%)	2021 N (%)	*p*-Value
Number of patients	36,076	23,903	
Number of patients treated with antibiotics	19,502 (54.06%)	14,660 (61.33%)	*p* < 0.00001 *
Gender			
Male	7872 (40.37%)	5753 (39.24%)	*p* < 0.05 *
Female	11,630 (59.63%)	14,255 (60.76%)	
Age in years			
Mean	54.15	53.72	*p* > 0.05 **
SD	20.64	20.66	
Outcome			
Died	902 (4.63%)	1179 (8.04%)	*p* < 0.05 *
Discharged	18,600 (95.37%)	13,481 (91.96%)	

* Pearson’s Chi-squared test, ** *t*-test.

**Table 2:** The distribution of clinical conditions (number of patients) for which antibiotics were prescribed.

Type of Infection/Condition	2019		2021		*p* *
Genito-urinary	2841	14.57%	1498	10.22%	<0.00001
Multiple localization	2384	12.22%	1877	12.80%	0.11
Wound	2092	10.73%	897	6.12%	<0.00001
Digestive	1126	5.77%	705	4.81%	<0.00001
Heart diseases	1384	7.10%	1050	7.16%	0.83
Respiratory	986	5.06%	815	5.56%	<0.05
Systemic	744	3.81%	565	3.85%	0.87
Surgical complications	213	1.09%	443	3.02%	<0.00001
COVID-19	0	0	109	0.74%	-
COVID-19 and associated bacterial infection	0	0	475	3.24%	-
Other viral aetiology	30	0.15%	298	2.03%	<0.00001
Osteoarthritis	49	0.25%	36	0.25%	0.99
Nervous system	11	0.06%	16	0.11%	0.13
Tuberculosis	5	0.03%	7	0.05%	0.43
Other conditions without infectious disease	1205	6.18%	651	4.44%	<0.00001
Prophylactic administration	6432	32.98%	5218	35.59%	<0.00001
Total	19,502		14,660		34,162

* Pearson’s Chi-squared test.

**Table 3:** Number of patients treated with antibiotics according to the type of ward.

Ward	2019 Number (%)	2021 Number (%)	*p* *
Surgical	11,453 (58.73)	7875 (53.72)	*p* < 0.00001
Medical	4462 (22.88)	2751 (18.77)	
Intensive care unit	3587 (18.39)	4034 (27.52)	

* Pearson’s Chi-squared test.

**Table 4:** Antibiotic consumption by grouped departments expressed as Antibiotic Consumption Index (ACI).

Ward	2019	2021	*p* *
Surgical	933.13 (56.06)	4421.46 (73.91)	<0.00001
Medical	350.21 (21.04)	1215.75 (20.34)	0.54
Intensive care unit	381.04 (22.89)	344.59 (5.76)	<0.00001

* Pearson’s Chi-squared test.

**Table 5:** Antibiotics prescribed according to AWaRe classification, expressed as ACI, in 2019 and 2021.

AWaRe Group	2019 Number (%)	2021 Number (%)	*p* *
Watch	1055.81 (63.44)	3287.62 (54.96)	<0.00001
Access	555.26 (33.36)	2500.74 (41.81)	
Reserve	19.26 (1.16)	47.83 (0.8)	

* Pearson’s Chi-squared test.

**Table 6:** Access group of antibiotics, expressed as ACI, in 2019 and 2021.

Access Group	2019	2021	*p* *
Metronidazole	104.81	1216.38	<0.00001
Amikacin	34.24	308.83	<0.00001
Ampicillin	132.46	297.06	<0.00001
Gentamicin	89.45	116.54	<0.00001
Clindamycin	42.85	89.07	<0.01
Benzylpenicillin	22.71	2.38	<0.00001
Amoxicillin/clavulanic acid	61.47	211.72	0.82
Amoxicillin	43.03	170.93	0.61
Doxycycline	8.76	54.61	0.16
Oxacillin	9.78	12.13	0.05
Ampicillin/sulbactam	5.33	11.71	0.51
Cefazolin	0.37	-	-

* Pearson’s Chi-squared test.

**Table 7:** Watch group of antibiotics, expressed as ACI, in 2019 and 2021.

Watch Group	2019	2021	*p* *
Ceftriaxone	450.90	1438.52	<0.05
Cefuroxime	255.52	449.36	<0.00001
Cefixime	9.95	434.70	<0.00001
Rifaximin	67.63	123.53	<0.00001
Teicoplanin	22.20	20.06	<0.00001
Ciprofloxacin	88.42	288.43	0.45
Meropenem	31.90	145.61	0.25
Ceftazidime	25.21	59.86	0.1
Cefoperazone	21.57	55.75	0.24
Vancomycin	18.22	57.63	0.73
Levofloxacin	10,54	59.81	0.21
Moxifloxacin	15.85	35.20	0.15
Clarithromycin	7.77	31.24	0.93
Ertapenem	4.81	18.11	1
Cefepime	2.34	19.09	0.34
Rifampicin	4.46	15.92	1
Piperacillin/tazobactam	3.36	10.29	1
Norfloxacin	3.17	7.07	0.74
Erythromycin	2.6	7.13	1
Fosfomycin	2.12	5.93	1
Imipenem/Cilastin	3.04	4.93	0.49
Ofloxacin	2.31	3.89	0.65
Azithromycin	0.25	4.87	0.69
Cefaclor	1.67	-	-

* Pearson’s Chi-squared test.

**Table 8:** Reserve group of antibiotics, expressed as ACI, in 2019 and 2021.

Reserve Group	2019	2021	*p* *
Colistin	8.98	21.54	0.42
Tigecycline	4.1	12.38	0.99
Linezolid	5.35	6.74	0.12
Ceftazidime/avibactam	0.83	6.91	0.75
Imipenem/cilastatin/relebactam	-	0.23	-

* Pearson’s Chi-squared test.

**Table 9:** Evaluation of antibiotic prescribed by class, expressed as ACI, in 2019 and 2021.

Class	2019	2021	*p* *
Cephems	767.53	2457.28	<0.001
Imidazoles	104.81	1216.38	<0.00001
Penicillins	207.98	482.50	<0.00001
Rifamycins	67.63	123.53	<0.00001
Lincosamides	42.85	89.07	<0.01
Glycopeptides	40.42	77.69	<0.01
Aminoglycosides	123.69	425.37	0.69
Quinolones	120.29	394.40	0.39
Beta-lactam/Inhibitors	105.05	386.32	0.87
Penems	39.75	168.88	0.38
Tetracyclines	12.86	67	0.27
Macrolides	10.62	43.24	0.84
Lipopeptides	8.98	21.54	0.41
Ansamycins	4.46	15.92	1
Oxazolidinones	5.35	6.75	0.12
Fosfomycin	2.12	5.93	1
Folate pathway inhibitors	-	-	-
Nitrofurans	-	-	-

* Pearson’s Chi-squared test.

**Table 10:** Sensitivity rate of the tested antibiotics.

Antibiotic Name	Cumulative Sensitivity Rate (%)		*p* *
	2019	2021	
Amikacin	84.68	78.39	<0.00001
Piperacillin/tazobactam	64.80	53.60	<0.00001
Ceftazidime/avibactam	71.86	46.82	<0.00001
Ceftazidime	58.40	55.69	<0.05
Cefepime	65.29	59.29	<0.00001
Cefotaxime	59.98	56.70	<0.05
Cefixime	44.40	36.00	<0.05
Trimethoprim/sulfamethoxazole	56.61	52.99	<0.01
Fosfomycin	88.99	91.39	<0.05
Vancomycin	79.57	88.79	<0.00001
Clindamycin	55.33	62.94	<0.01
Colistin	71.70	81.26	<0.00001
Imipenem	65.22	56.32	<0.00001
Meropenem	74.01	60.50	<0.00001
Ertapenem	84.10	77.42	<0.00001
Penicillin G	38.08	26.02	<0.00001
Azithromycin	50.00	20.83	<0.05
Ciprofloxacin	48.91	42.59	<0.00001
Levofloxacin	49.40	43.00	<0.05
Ofloxacin	51.19	67.84	<0.001
Gentamicin	65.60	66.50	0.39
Tobramycin	37.58	32.97	0.06
Rifampin	82.89	88.04	0.056
Amoxicillin/clavulanic acid	45.99	44.20	0.22
Ampicillin/sulbactam	41.51	38.96	0.61
Cefoperazone	58.31	51.19	0.28
Ceftriaxone	52.57	49.83	0.34
Cefuroxime	58.06	57.40	0.56
Teicoplanin	92.14	93.97	0.13
Erythromycin	32.33	31.09	0.55
Nitrofurantoin	61.19	63.81	0.12
Linezolid	91.54	91.56	1
Ampicillin	27.02	28.30	0.32
Oxacillin	35.07	33.13	0.61
Moxifloxacin	73.99	71.85	0.35
Tetracycline	34.04	38.56	0.07

* Pearson’s Chi-squared test.

**Table 11:** Number of isolated ESKAPE pathogens and their resistance phenotype.

Strain/Resistance Phenotype	2019	2021	*p* *
*Acinetobacter baumannii*	501	751	
AB-CR	365	691	<0.00001
*Enterobacter* sp.	142	126	
E-ESBL	42	62	<0.01
E-CR	5	15	<0.05
*Escherichia coli*	1368	1261	
EC-ESBL	218	302	<0.00001
EC-CR	41	63	<0.05
*Enterococcus faecium*	140	178	
VRE	46	60	0.9681
*Klebsiella pneumoniae*	833	1039	
KP-ESBL	450	571	0.721
KP-CR	333	520	<0.00001
*Pseudomonas aeruginosa*	521	575	
PA-CR	219	299	<0.01
*Staphylococcus aureus*	626	312	
MRSA	426	125	<0.00001

* Pearson’s Chi-squared test. Abbreviations: AB—*Acinetobacter baumannii*, E—*Enterobacter* sp., EC—*Escherichia coli*, KP—*Klebsiella pneumoniae*, PA—*Pseudomonas aeruginosa*, CR—carbapenem-resistant, ESBL—Extended-Spectrum β-Lactamases, VRE—vancomycin-resistant enterococci, MRSA—Methicillin-resistant *Staphylococcus aureus*.

## Data Availability

Data are contained within the article.
